# Techniques and Strategies in Drug Design and Discovery

**DOI:** 10.3390/ijms25031364

**Published:** 2024-01-23

**Authors:** George Mihai Nitulescu

**Affiliations:** Faculty of Pharmacy, “Carol Davila” University of Medicine and Pharmacy, 6 Traian Vuia Street, 020956 Bucharest, Romania; george.nitulescu@umfcd.ro

The process of drug discovery constitutes a highly intricate and formidable undertaking, encompassing the identification and advancement of novel therapeutic entities. In recent epochs, the procedural landscape of drug discovery has undergone rapid evolution, incorporating a multitude of paradigms and techniques [1,2,3]. Nevertheless, the absence of an infallible algorithm for assured success persists. Despite extensive research endeavors, fortuitous discoveries, and the extraordinary exercise of intuition, inspiration, and creativity by researchers, a pivotal role endures for these qualities in the realm of drug discovery, which continues to command substantial interest among the scientific community [4,5]. The imperatives of novelty and ingenuity are indispensable to contend with the prevailing challenge of cost efficiency [6,7].

This Special Issue was focused on providing an extensive and diverse platform for researchers to expound upon their contemporary efforts aimed to refine existing methodologies with the objective of discovering new molecular entities. The thematic objective was directed towards presenting new research and comprehensive reviews that elucidated prevailing practices across all aspects of drug discovery and drug development.

The scaffold-based method in drug design is a strategic approach that focuses on identifying and utilizing bioactive scaffolds associated with specific biological activities as templates for the design of new compounds [8,9,10,11]. Tamanova et al. [12] used a scaffold-based approach to devise novel 2,7,9-trisubstituted 8-oxopurines as FMS-like tyrosine kinase 3 (FLT3) inhibitors and analyzed their structure–activity relationship studies in their effort to find better solutions for the management of FLT3-positive acute myeloid leukemia [13]. Their findings demonstrated that substituents at positions 7 and 9 exert a modulating effect on the activity between CDK4 and FLT3 kinase, with the isopropyl group at position 7 significantly enhancing selectivity toward FLT3. This enhancement led to the identification of 9-cyclopentyl-7-isopropyl-2-((4-(piperazin-1-yl)phenyl)amino)-7,9-dihydro-8H-purin-8-one. Cellular analyses conducted in MV4-11 cells revealed the inhibition of FLT3 kinase autophosphorylation at nanomolar doses, encompassing the suppression of downstream STAT5 and ERK1/2 phosphorylation. Mechanistic studies in cell lines and activity observed in a mouse xenograft model in vivo are also detailed. Stecoza et al. [14] used a similar scaffold-based technique to develop new 1,3,4-thiadiazol-2-amine derivatives as anti-proliferative agents. The use of in silico methods like the PASS (Prediction of Activity Spectra for Substances) application [15,16] and docking studies indicated STAT3, Mcl-1, and CDK9 as the most likely mechanisms responsible for the anti-cancer effects. This work supports the importance of the 1,3,4-thiadiazol-2-amine scaffold in anti-cancer design [17,18].

The work of Lu Wang et al. [19] focused on developing a method relatively similar to PASS in order to identify potential drug–target interactions (DTIs). The use of these computational methods can systematically assess and prioritize the most probable targets for a specific drug [20,21,22,23], facilitating more focused experimental validation and uncovering new therapeutic uses for existing drugs [24,25]. Their study introduced AMMVF-DTI, an innovative end-to-end deep learning model employing an attention mechanism and multi-view fusion. AMMVF-DTI utilizes a multi-head self-attention mechanism to assess varying degrees of interaction between drugs and target proteins. It extracts interactive features from both node-level and graph-level embeddings, enhancing DTI modeling by incorporating local and global structures. Compared to state-of-the-art methods, AMMVF-DTI exhibits exceptional performance on human, *C. elegans*, and DrugBank baseline datasets. Deep learning techniques based on self-attention have shown promising applications in drug design [26,27,28] and were also used by the team of Ghemire et al. [29] to predict drug–target affinity (DTA). In the initial phases of drug development, the accurate prediction of drug–target affinity assumes paramount significance. This study introduced a model named Convolutional Self-Attention for Drug-Target Affinity (CSatDTA), employing convolution-based self-attention mechanisms on molecular drug and target sequences. CSatDTA demonstrated enhanced effectiveness in predicting DTA compared to previous convolution methods, which are constrained by notable limitations in this context. Unlike conventional convolutional neural networks (CNNs) that focus on specific information regions [30], thereby missing comprehensive details, CSatDTA integrates self-attention, a recent technique adept at capturing long-range interactions [31]. Comparative experiments underscore CSatDTA’s superiority over preceding sequence-based approaches, affirming its exceptional retention capabilities [29].

The review of Mercurio et al. [32] is based on another important strategy of drug design, the target-based approach [33,34]. Sam (Sterile alpha motif) domains are versatile protein binding modules found in various organisms. The EphA2 receptor’s C-terminal Sam domain (EphA2-Sam) interacts with regulators like Ship2 and Odin, influencing receptor stability. Ship2 promotes pro-oncogenic outcomes by reducing EphA2 endocytosis, while Odin hinders ubiquitination, contributing to stability. With EphA2 overexpression in tumors, inhibiting Sam–Sam interactions via peptides could be an anti-cancer strategy. The review explored the structural–functional aspects of EphA2-Sam and its interactome, summarizing design strategies for peptides targeting these interactions and arguing that efforts are needed to enhance peptide affinities toward Sam domains in order to increase their anti-cancer potential [32].

The articles published in this Special Issue present the successful use of diverse strategies of drug design, integrating scaffold-based, deep learning, and target-based approaches. Each method contributes unique insights and advancements, emphasizing the continual need for innovation and refinement in the pursuit of effective and targeted drug development.
